# Serum and Hepatic Autofluorescence as a Real-Time Diagnostic Tool for Early Cholestasis Assessment

**DOI:** 10.3390/ijms19092634

**Published:** 2018-09-05

**Authors:** Anna C. Croce, Giovanni Bottiroli, Laura G. Di Pasqua, Clarissa Berardo, Veronica Siciliano, Vittoria Rizzo, Mariapia Vairetti, Andrea Ferrigno

**Affiliations:** 1 Institute of Molecular Genetics, Italian National Research Council (CNR), Via Abbiategrasso 207, I-27100 Pavia, Italy; croce@igm.cnr.it (A.C.C.); bottiroli@igm.cnr.it (G.B.); 2 Department of Biology & Biotechnology, University of Pavia, Via Ferrata 9, I-27100 Pavia, Italy; 3 Department of Internal Medicine and Therapeutics, University of Pavia, Via Ferrata 9, I-27100 Pavia, Italy; lauragiuseppin.dipasqua01@universitadipavia.it (L.G.D.P.); clarissa.berardo01@universitadipavia.it (C.B.); veronica.siciliano01@universitadipavia.it (V.S.); mariapia.vairetti@unipv.it (M.V.); 4 Department of Molecular Medicine, IRCCS San Matteo, University of Pavia, Viale Golgi 5, I-27100 Pavia, Italy; v.rizzo@smatteo.pv.it

**Keywords:** BDL model, extrahepatic cholestasis, bilirubin, endogenous porphyrins, energy metabolism, oxidative stress, spectrofluorometry

## Abstract

While it is well established that various factors can impair the production and flow of bile and lead to cholestatic disease in hepatic and extrahepatic sites, an enhanced assessment of the biomarkers of the underlying pathophysiological mechanisms is still needed to improve early diagnosis and therapeutic strategies. Hence, we investigated fluorescing endogenous biomolecules as possible intrinsic biomarkers of molecular and cellular changes in cholestasis. Spectroscopic autofluorescence (AF) analysis was performed using a fiber optic probe (366 nm excitation), under living conditions and in serum, on the livers of male Wistar rats submitted to bile duct ligation (BDL, 24, 48, and 72 h). Biomarkers of liver injury were assayed biochemically. In the serum, AF analysis distinctly detected increased bilirubin at 24 h BDL. A continuous, significant increase in red-fluorescing porphyrin derivatives indicated the subversion of heme metabolism, consistent with an almost twofold increase in the serum iron at 72 h BDL. In the liver, changes in the AF of NAD(P)H and flavins, as well as lipopigments, indicated the impairment of mitochondrial functionality, oxidative stress, and the accumulation of oxidative products. A serum/hepatic AF profile can be thus proposed as a supportive diagnostic tool for the in situ, real-time study of bio-metabolic alterations in bile duct ligation (BDL) in experimental hepatology, with the potential to eventually translate to clinical diagnosis.

## 1. Introduction

Various factors can impair the production and flow of bile and subsequently lead to cholestatic disease. Adverse effects at both hepatic and extrahepatic sites highlight the clinical significance of cholestatic disease, as well as the need to further investigate the key biomarkers of the complex molecular mechanisms underlying its pathophysiology, to better support diagnosis and develop new therapeutic strategies [1,2].

BDL in rodents is a model of extrahepatic biliary obstruction, used to investigate both liver injury and associated extrahepatic disease, such as cholemic nephropathy or encephalopathy [3,4,5,6,7]. BDL induces a cascade of an ensuing, dynamic series of acute and chronic injuries [5,8,9]. The early effects on liver bio-metabolic features may include dysfunction of mitochondria and oxidative stress [10,11,12] and activation of the inflammatory response of the liver tissue, as well as an increase in the proliferation of hepatocellular or biliary epithelial cells and in mediators of collagen production and fibrosis. Fibrosis, in turn, may well progress to cirrhosis. Besides increasing levels of serum bilirubin, liver disease progression can be preceded or accompanied by the increased expression of various growth factors, such as alanine aminotransferase (ALT) or alkaline phosphatase (AP), respectively reflecting hepatocellular injury or de novo synthesis in hepatic tissue [8,9].

About 1 week after BDL, fibrosis can be clearly detected histologically [8,13]. For this reason, BDL has been recently used as a model of liver fibrosis to validate an automated system to improve the staging and monitoring of collagen deposition as a diagnostic support in experimental pharmacology. This system is based on the intrinsic optical properties of collagen, consisting of AF or anisotropy with the emission of light by a second-harmonic generation, to detect fibrosis in a label-free manner [14]. However, the various and complex bio-metabolic features of the liver involve additional, different endogenous biomolecules, able to fluoresce in the UV–visible interval when excited with light at a suitable wavelength. These endogenous fluorophores can contribute to a varying extent to the overall AF signal, depending on their amount and intrinsic emission properties, which in turn also depend on the involvement of these biomolecules in the metabolic processes or tissue architecture under normal or diseased conditions [15].

Using the AF signal in optical biopsy to estimate the contribution of individual endogenous fluorophores has been, therefore, promoted as a valuable tool for the real-time detection and characterization of molecular and bio-metabolic changes in hepatology [16].

The aim of our work is thus to apply fiber optic probe AF analysis to the investigation of endogenous fluorophores, as possible intrinsic molecular and cellular biomarkers of changes in the liver and in the serum in an early BDL model, for advanced application in experimental hepatology and possible translation to a clinic setting.

## 2. Results

### 2.1. Serum Analysis

In our BDL model, biochemical assays confirmed a highly significant increase in the levels of aspartate transaminase (AST), ALT, γ-glutamyltransferase (γGT), and AP, the common serum biomarkers of liver injury, and of iron, a byproduct of heme degradation related to changes in heme-oxygenase activity. BDL also affected uric acid and urea, significant for kidney dysfunction (Table 1).

**Figure 1:**
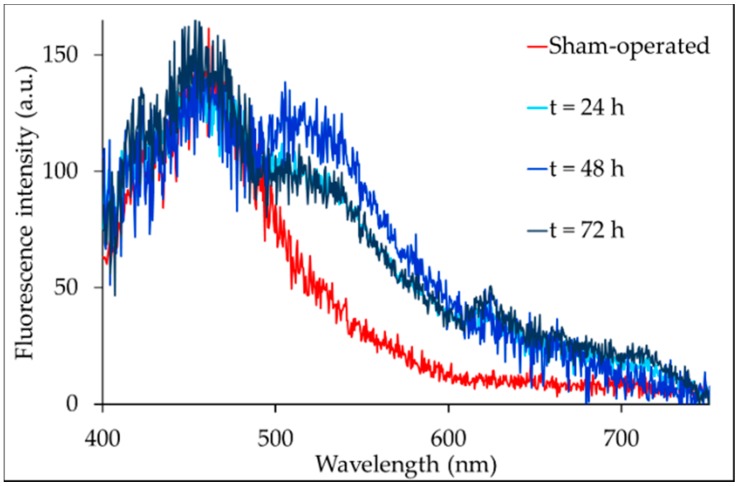
Examples of autofluorescence (AF) spectra measured via fiber optic probe in the serum from sham-operated or BDL rats. To better appreciate the shoulder in the 500–570 nm range, the spectra are normalized to the AF amplitude at the crossover point (485 nm) with the main band in the blue region.

The serum samples submitted to spectroscopy analysis showed well-defined AF spectra (Figure 1). In particular, the serum AF emission consisted of one major band, peaking at about 455 nm in the sham-operated rats. 

The spectra of the serum from the BDL rats showed this major band superimposed by a shoulder in the 500–570 nm interval, the spectral region typical of bilirubin [17].

The bichromophore nature of bilirubin causes its emission to be composed by two bands, a predominant band centered at about 517–530 nm and a secondary band centered at about 570 nm. 

The conformation of the bilirubin molecule and its dependence on the microenvironment may affect the balance between the two bands and, consequently, the ratio between their respective area values [17]. In our serum samples, the relative contribution of the two bands to the overall AF emission was estimated by spectral fitting analysis (Figure S1).

The results are summarized in Table 2 along with the (517–530 nm)/(570 nm) ratios, which indicate no major variations in the balance of the two bands between the samples taken at different BDL times (Table 2).

**Figure 2:**
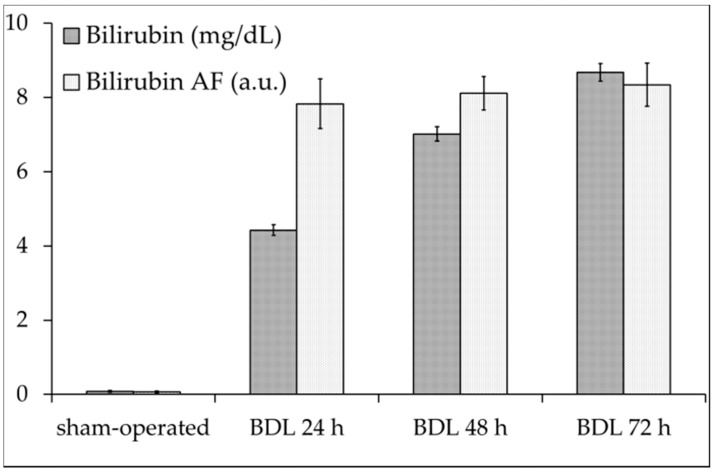
The serum bilirubin analysis at increasing times after BDL, the total concentration of bilirubin assayed biochemically (mg/dL), and the bilirubin AF values (a.u.) estimated by curve fitting analysis. The real AF values were calculated from the relative contribution of the 517–530 nm–570 nm shoulder to the overall integrated spectral area, corrected for the measured emission values of the overall serum spectra (4490 ± 251, 3720 ± 287, 4390 ± 219, 4650 ± 155 a.u., for sham-operated rats and 24, 48, 72 h BDL rats, respectively), and divided by 100 for the convenience of presentation. Mean values ± S.E. (*n* = 5 liver/group); *p* < 0.001: sham-operated vs. all BDL times, for both the bilirubin concentration and AF values, and 24 h BDL vs. 48 h and 72 h BDL (biochemical concentration and AF); *p* < 0.01 48 h BDL vs. 72 h BDL (biochemical concentration).

The sum of each pair of bilirubin AF data was then used to determine the emission spectral area values, represented in Figure 2, along with the conventional biochemical assay results.

Compared with that of the sham-operated rats, the total AF emission area relatable to bilirubin showed a marked increase at 24 h BDL, similar to the biochemical assay, whereas the bilirubin signal only showed a small increase at 48 and 72 h BDL.

To investigate the different behavior between the AF and biochemical data, we considered the molar concentration of bilirubin. This was about 6 × 10^−7^ M in the sham-operated rats and within the 10^−5^ M range for the BDL rats.

Because the calibration of the bilirubin AF versus the concentration in the serum can be affected by changes in the quantum yield due to the interaction of the bichromophore with its various components, the AF response of bilirubin was calibrated in a solution of hexadecyltrimethylammonium-bromide (CTAB) 1 × 10^−3^ M. Here, the bilirubin AF was detectable starting from a concentration of 1 × 10^−6^ M, while the AF signal saturated at concentration higher than 5 × 10^−5^ M.

In addition to the bilirubin AF, our attention was attracted by the minor bands occurring in the red emission region (Figure 1). The position of these bands is typical of porphyrin derivatives [18] and favors their discrimination from the long-wavelength tail of the spectrum by curve fitting analysis.

Hence, the red signal’s contribution to the overall AF emission was estimated and underscored the marked increase of porphyrins already at 24 h BDL (Table 2).

Notably, the highest level was detected at 72 h of BDL, in parallel with an almost twofold increase in the serum iron concentration in the BDL versus the sham-operated rats (Table 1).

### 2.2. Liver Autofluorescence

**Figure 3:**
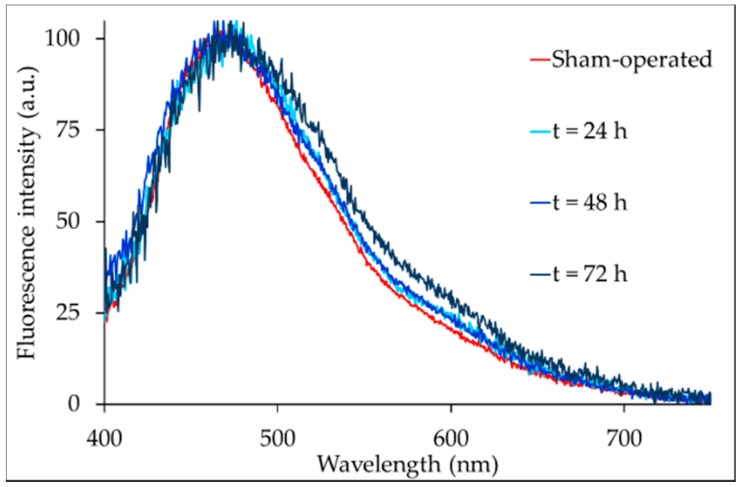
Examples of the AF spectra collected via the fiber optic probe from the livers of sham-operated or BDL rats. For easier appreciation of the changes in the spectral shape during BDL time, the spectra have been normalized to 100 a.u. at the peak maximum value.

The liver AF spectra consisted of a main band covering the 400–700 nm interval (Figure 3).

As the BDL time lengthened, the AF spectra widened towards longer wavelengths (Figure 3 and Figure S2).

The spectral changes reflected variations in the relative contribution of the various endogenous fluorophores to the overall AF signal, estimated through spectral fitting analysis (Table 3; Figure S2).

The fitting analysis underscored the continuous increase in the contributions of proteins and lipopigments. Flavins were also found to increase, but only between sham-operated and 24 h BDL rats, as they subsequently remained at that level at 48 and 72 h BDL. On the contrary, vitamin A, fatty acids, and NAD(P)H, in particular the bound form, all decreased.

The autofluorescence data indicated also an increased presence of proteins and lipopigments, the latter indicating a gathering accumulation of oxidative event products.

The changes in NAD(P)H and flavins drew our attention to the liver energy metabolism. Considering the essential involvement of NAD(P)H and flavins as coenzymes in the biomolecular pathways of energy production, their AF data were further processed and compared with the adenosine triphosphate/adenosine diphosphate (ATP/ADP) ratios, the conventional biochemical markers of tissue energy status (Table 4).

The results showed that at 72 h BDL, both the NAD(P)H_free_/NAD(P)H_bound_ ratios and the optical redox ratios were markedly increased, in parallel with the respective significant decrease of the ATP/ADT ratio and increase in thiobarbituric acid reactive substances (TBARS). The latter, in turn, are consistent with the significant increase of lipopigments as compared with the sham-operated rats.

## 3. Discussion

Bile duct ligation (BDL) is an established and reproducible procedure of experimental extrahepatic cholestasis. Given the central role played by the liver in many body functions, from the maintenance of systemic metabolic and detoxification functions, to immunological tasks and the regulation of blood oncotic pressure and hemostasis, the use of the animal model is virtually obligatory for various purposes of investigation in experimental hepatology [19,20,21]. This includes investigations of molecular and biochemical changes to assess pathophysiological mechanisms of BDL injury in hepatic and extrahepatic sites, to identify new diagnostic biomarkers, and to develop and improve diagnostic procedures and therapeutic approaches. Because BDL causes a sequential progression from acute to chronic cholestatic injury, the BDL model is particularly suitable for a time-resolved study of specifically related biomarkers [9]. In this view, real-time in situ AF analysis is a promising tool with regards to improving the investigation of early metabolic changes [16]. In the present work, the induction of liver injury was confirmed by an increase in the serum levels of conventional hepatic biomarkers such as AST, ALT, γGT, and AP, assayed biochemically. Changes in uric acid and urea levels were also found, indicating initial renal consequences [3,22,23]. Conventional serum biochemistry also confirmed the increase in bilirubin, in particular the conjugated form, very much in keeping with the literature for both animal models and human extrahepatic cholestasis [24,25].

The autofluorescence analysis of serum evidenced a main emission band in the blue spectral region, very likely ascribable to albumin. In fact, albumin is a main component of serum, and its remarkable ability to be excited and fluoresce in the UV range may account for its detection under the slightly longer excitation at 366 nm. In the BDL serum samples the main, blue band was over-imposed by a shoulder in the 517–570 nm spectral region. The identification of this shoulder with bilirubin emission is in keeping with previous reports regarding bilirubin’s fluorescence properties [17,26]. In particular, bilirubin acts as a bichromophore, because it consists of two covalently linked chromophores that give rise to two emission bands: a main band centered at about 517–530 nm and a secondary band, centered at about 570 nm. The excitation energy can be exchanged between the two chromophores that make up the two chromophores to fluoresce with different yields, depending on the bilirubin’s molecular conformation and its sensitivity to its own concentration and microenvironment. As a result, the balance between the two bands can vary, as previously demonstrated for unconjugated bilirubin in the presence of the cationic detergent CTAB in micellar or premicellar concentrations, in an aqueous solution. The area values of the 517–530 nm and 570 nm bands were estimated by spectral fitting analysis, and the ratios between them were 4.22 and 7.38, respectively [17].

In our serum samples, the spectral shape remained almost unchanged, as indicated by the (517–530 nm)/(570 nm) ratios ranging around 4.00. This finding can be explained by two factors: (i) the prevalence of direct, conjugated bilirubin measured by biochemical assay (72 h BDL, direct and indirect bilirubin: 6.99 ± 0.15 vs. 1.69 ± 0.82 mg/dL, respectively), in keeping with the literature [25]; and (ii) the lesser sensitivity to the microenvironment of conjugated bilirubin as compared with the unconjugated form. In fact, pure taurobilirubin was used to represent the conjugated, water-soluble bilirubin, showed no great changes in the (517–530 nm)/(570 nm) ratio, which was around 4.00 regardless of micellar or pre-micellar CTAB concentrations.

As to the overall bilirubin AF emission, it must be emphasized that the almost negligible signal in sham-operated rats rose to a contribution to the overall emission spectrum ranging from 17% to 21% in the BDL samples. As compared with the biochemical assay data, the much higher response of the AF at 24 h BDL than that between 48 and 72 h BDL may reflect the bilirubin AF saturation at concentrations greater than 5 × 10^−5^ M. This finding, however, does not invalidate the diagnostic role of the bilirubin AF, which is expected to improve the time-effective detection of the early increase in serum bilirubin.

The analysis of the serum AF also revealed a remarkable rising in the red signal ascribable to porphyrin derivatives at BDL 72 h, in parallel with a very significant increase in serum iron concentration. In general, the increase in porphyrin derivatives in the blood may reflect systemic or hepatic subversion in heme and iron metabolism for a variety of reasons, including porphyria, liver viral infection, or the presence of tumors [18,27,28,29]. With more specific reference to BDL, we may recall reports on the consequences on the expression of heme oxygenase (HO-1), along with the related heme metabolism and production of iron and carbon monoxide as byproducts of heme degradation [30], and on the heme-containing cytochrome P450 [9,31], all of which draw attention to the fluorescence of porphyrins in the serum as a possible early biomarker of injury in extrahepatic cholestasis.

In the liver tissue, BDL caused an increase in the protein AF emission signal. This finding is strongly consistent with numerous reports from the literature, in keeping with the ability of fibrous proteins to give rise to a much more remarkable AF emission in the near-UV-blue region as compared with globular proteins [16,32,33,34]. Hence, our results are consistent with the well-recognized ability of BDL to induce fibrosis [13,35], which is preceded by changes in the extracellular matrix, as we documented in 3-day BDL rats by matrix metalloproteinases activation [36]. The ability of AF to sense the deposition of the fibrillary precursors of collagen earlier than the detection of fibrosis by more conventional procedures [14] is also corroborated by our previous findings on the in vivo detection of the increase in the protein AF before collagen detection by conventional Sirius red staining in a methyl choline deficient (MCD) diet model of a fatty liver with oxidative stress [37].

The increase in the lipofuscin-like lipopigments AF reflects the presence of oxidative products [38,39]. The comparison between *t* = 0 and *t* = 72 h data revealed an approximately 45% increase for the AF data, much higher than the roughly 25% increase for biochemical TBARS levels. This finding may be explained considering that lipofuscin-like lipopigments represent the enduring accumulation of oxidation products of various kinds of biomolecules, including carotenoids and porphyrins in addition to lipids and proteins as their main constituents, fully reflecting the gathering of oxidative events. On the contrary, the TBARS assay is based on the detection of malondialdehyde, as a short-lived thiobarbituric acid reactive substance in a window of time during the course of lipid peroxidation. Our AF results are consistent with the increase in liver oxidative stress in cholestasis [10], with the advantage of detecting previous oxidative events.

The NAD(P)H and flavin AF, measured in vivo by the fiber optic probe, provided parameters for assessing the metabolic and energy status of cells and tissues. The NAD(P)H_total_ AF levels likely reflect the reserve of reductive energy, while the optical redox ratio and the NAD(P)H_bound/free_ ratio are specifically related to mitochondrial integrity and functionality [40,41,42,43]. Indeed, the obligatory interaction between the coenzyme NAD(P)H and its related enzymes involves more numerous binding sites along the aerobic pathways of energy production than it does along the anaerobic pathways. In addition, the mitochondrial flavoproteins participating in respiratory functions account for at least 90% of the overall flavin AF emissions from the liver tissue [44,45]. On this basis, our data on NAD(P)H and NAD(P)H_bound/free_ ratios clearly indicated that the 72 h after BDL resulted in a decrease both in the reductive energy reserve and in the mitochondrial functionality in the liver tissue. The optical redox ratio, in turn, indicated an increased oxidized state, in keeping with the increase in the accumulation of oxidized species revealed by the AF of lipopigments. These indications are fully consistent with reports on the increased enrollment of glycolytic energy metabolism, which sustains energy production to compensate for mitochondrial dysfunction. They are also in keeping with the occurrence of mitochondria dysfunction and oxidative stress in the development of injury in hepatic and extrahepatic sites in cholestasis [4,11,12].

## 4. Materials and Methods

### 4.1. Animal Model

The experimental protocol was approved by the Italian Ministry of Health and the University Commission for Animal Care (Document 3/2012, 25 February 2012), and the animals were cared for in accordance with its guidelines. Male Wistar rats (250–300 g, Harlan-Nossan, Milano, Italy) had free access to water and food. A total of 3 groups underwent BDL 24, 48 and 72 h (*n* = 5 each group). In the BDL model, the abdomen was opened by a median incision in pentobarbital-anesthetized rats (50 mg/kg), and the common bile duct was double ligated and cut between the ligatures (BDL). The sham-operated rats underwent similar manipulation but with no bile duct ligation. At the BDL time points indicated, the rats were anesthetized, and the liver was exposed for the AF measurements. Blood samples were then collected from the abdominal aorta and immediately centrifuged to separate the serum. Hepatic biopsies from the left lobe were also collected and immediately snap-frozen in liquid nitrogen for subsequent biochemical assays. To prevent heat loss, the rats were maintained on a warm support during the procedures.

### 4.2. Chemicals and Biochemical Assays

Ditaurobilirubin (Bilirubin conjugated; Lee Biosolutions, St. Louis, MO, USA) was prepared as a stock solution 1 × 10^−3^ in phosphate-buffered saline (PBS), pH 7.4, to be diluted with hexadecyltrimethylammonium-bromide (CTAB, stock solution 1 × 10^−1^ M in PBS) to obtain the final concentrations as follows: Ditaurobilirubin 1 × 10^−5^ M, in CTAB, 1 × 10^−4^ M or 1 × 10^−3^ M. These were, respectively, the pre- and supra micellar concentrations [46].

The serum levels of alanine transaminase (ALT), aspartate transaminase (AST), alkaline phosphatase (AP), γ-glutamyltransferase (γGT), uric acid, urea, iron, and total and direct bilirubin were measured by a Hitachi 747 analyzer (Roche/Hitachi, Indianapolis, IN, USA). Indirect bilirubin was quantified as the difference between the total and direct bilirubin.

The hepatic proteins were measured according to Lowry’s method, using bovine serum albumin as the standard [47]. The levels of liver lipid peroxidation in terms of thiobarbituric acid reactive substances (TBARS) were determined as previously described [36,48]. The liver tissue ATP was measured by the luminescence method using the ATP-lite luciferin/luciferase kit (Perkin Elmer Inc., Waltham, MA, USA). The luminescence was evaluated on a Perkin Elmer Victor II, using a white 96-well plate [49]. Unless otherwise stated, the chemicals were purchased from Sigma Chem. Co. (St. Louis, MO, USA).

### 4.3. Autofluorescence Spectrofluorometric Analysis

The autofluorescence spectra were obtained with an optical multichannel analyzer (OMA, Hamamatsu Photonics Deutschland GmbH, Herrsching am Ammersee, Germany. Mod. PMA11) coupled with the fiber optic probe by means of a beam splitting module (Oriel Instruments, Stradford, CT, USA), mounting a 390 nm dichroic mirror (Chroma Technology Corp., Rockingham, VT, USA), and previously described in detail [17]. The module delivered the excitation light (LED source, 366 nm, 5.0 W; SestoSensor, Bologna, Italy) to the front of a single optic fiber (300 µm diameter), which guided both the excitation light to the measuring site and the resultant AF emission to the module, from which a 17-fiber bundle (200 µm diameter each) delivered the AF light to the OMA detector. The emission spectra were recorded in the 400–750 nm range. The excitation shutter opened automatically before each measurement. Each spectral acquisition set consisted of 10 sequential scans (400 ms each, for a total measuring time of 4 s). The autofluorescence spectra from the livers were collected under living conditions by gently inserting the fiber optic probe into the liver tissue. All the measurements were taken from the left lobe, for homogeneity with the biochemical data. The spectra with appreciable alterations of emission profile indicating deoxyhaemoglobin reabsorption were discarded. The same device was used to measure the serum AF by immersing the fiber probe directly inside the vial where the fluid was collected. The measurements were performed on fresh serum, paying attention to preserve it from light to avoid photobleaching effects.

### 4.4. Spectral Fitting Analysis

The autofluorescence spectra were analyzed by means of a curve-fitting procedure, to estimate the relative contribution of each fluorophore to the overall signal. Before analysis, the peak amplitude was normalized to 100, and the wavelengths were converted into wave-number units, supposing an in-homogenous line broadening in the frequency. The analysis was performed using half-Gaussian modified Gaussian spectral functions (GMG), each one representing the emission profile of a fluorophore expected to contribute to the overall emission signal. The parameters of the GMG functions were already derived from the emissions of pure compounds in order to correctly identify and provide an optical quantification of these biomolecules according to our prior works [18,50]. The analysis was performed by means of an iterative non-linear curve-fitting procedure (PeakFit; SPSS Science, Chicago, IL, USA) based on the Marquardt–Levenberg algorithm [51] and on the finding of the true absolute minimum value of the sum of squared deviations (χ^2^). Subsequent adjustments of the combination of the spectral functions representing each endogenous fluorophore were performed, until the curve resulting from their sum gave the best match with the experimental spectrum profile. The goodness of fit was verified in terms of the residuals and r^2^ coefficient of determination.

### 4.5. Statistical Analysis

Power analysis was used to calculate group size. The sample size was the average of the values obtained by imposing an effect size ranging from 0.7 and 0.9, a power of 90%, and significance of 0.05. The calculations were performed using R Statistical Software (PWR package, version 3.5.1). The results are expressed as mean ± standard error (S.E.). The statistical analysis was performed by ANOVA and Tukey’s test for multiple comparisons.

## 5. Conclusions

The autofluorescence profiling of serum and the liver can provide much information on the molecular and cellular metabolism effects induced by BDL. Our findings further promote the application of AF-based optical diagnostic procedures as a valuable, supportive diagnostic tool in experimental hepatology on cholestasis, which will hopefully lead to its translation to clinical applications in the very near future.

## Figures and Tables

**Table 1:** Serum biochemical analysis in rats 72 h after after bile duct ligation (BDL) and in sham-operated rats.

Rat Model	AST (mU/mL) ^a^	ALT (mU/mL) ^a^	γGT (mU/mL) ^a^	AP (mU/mL) ^b^	Iron (mg/dL) ^a^	Uric Acid (mg/dL) ^b^	Urea (mg/dL) ^a^
Sham-operated	90.20 ± 7.10	36.41 ± 2.20	3.7 ± 0.02	259.63 ± 13.01	158.62 ± 18.01	1.15 ± 0.10	20.4 ± 0.9
BDL	636.22 ± 82.10	306.21 ± 35.10	10.21 ± 2.1	329.83 ± 25.04	294.22 ± 48.13	0.82 ± 0.05 ^b^	31.42 ± 1.25

Means ± standard error (S.E.), *n* = 5 livers per group. Statistical analysis: ^a^, *p* < 0.001; ^b^, *p* < 0.05 in the same column. AST: aspartate transaminase; ALT: alanine aminotransferase; γGT: γ-glutamyltransferase; AP: alkaline phosphatase.

**Table 2:** Curve fitting analysis of the serum AF spectra. The relative contribution of each selected endogenous fluorophore to the overall emission signal (400–750 nm range) was estimated from spectral areas after normalization to 100 a.u. (arbitrary unit).

BDL Time	Autofluorescence Signal Contributors				
	Blue Band (center = 455 nm)	Bilirubin (517–530 nm)	Bilirubin (570 nm)	Bilirubin (517–530 nm)/(570 nm)	Red Bands (600–770 nm)
Sham-operated	98.56 ± 5.26	≈0.09 ^§^	≈0.06 ^§^	§	1.76 ± 0.12 ^a^
*t* = 24 h	69.05 ± 3.49	16.98 ± 1.45	4.67 ± 0.34	4.17 ± 0.29	7.12 ± 0.40 ^b^
*t* = 48 h	70.22 ± 4.41	14.80 ± 0.76	3.68 ± 0.20	4.01 ± 0.31	8.71 ± 0.46 ^c^
*t* = 72 h	67.31 ± 4.77	14.31 ± 0.92	3.56 ± 0.24	4.02 ± 0.20	13.60 ± 0.62 ^b,c^

Values reported as means ± S.E., *n* = 5 livers per group. Statistical analysis: ^a^, *p* ≤ 0.001 versus all other data in the same column; ^b^, ^c^, *p* ≤ 0.001 in the same column. ^§^ Bilirubin ratio not calculated, due to the very low, unreliable 517–530 nm, 570 nm data.

**Table 3:** Curve fitting analysis of the liver tissue AF spectra. The relative contribution of each endogenous fluorophore to the overall emission signal in the 400–750 nm range was estimated from the spectral areas after normalization to 100 a.u.

BDL Time	Autofluorescence Endogenous Fluorophores						
	Proteins	NAD(P)H_bound_	NAD(P)H_free_	Flavins	Vitamin A	Fatty Acids	Lpgs
Sham-operated	3.72 ± 0.26 ^a,b^	13.06 ± 0.81 ^c^	39.23 ± 2.35	3.94 ± 0.20 ^d^	21.07 ± 1.07 ^a^	12.43 ± 0.71	6.01 ± 0.43 ^c^
*t* = 24 h	5.40 ± 0.44 ^a,a′^	11.76 ± 0.83	38.19 ± 2.29	5.02 ± 0.25	20.44 ± 1.22	11.42 ± 0.78	6.69 ± 0.55
*t* = 48 h	7.09 ± 0.49 ^b^	11.33 ± 0.68	37.88 ± 1.97	5.37 ± 0.26	18.35 ± 0.91	11.10 ± 0.59	6.98 ± 0.50
*t* = 72 h	8.80 ± 0.52 ^a′,b^	9.27 ± 0.46 ^c^	37.15 ± 1.67	5.02 ± 0.27	16.97 ± 0.69 ^a^	10.9 ± 0.46	8.78 ± 0.42 ^c^

Values reported as means ± S.E., 3 measurements for each of 5 livers per group. Statistical analysis: ^a^, ^a′^, *p* ≤ 0.01; ^b^, *p* ≤ 0.001; ^c^, *p* < 0.005 within marked data in the same column; ^d^, *p* ≤ 0.01 versus all other data in the same column. Lpgs: lipopigments.

**Table 4:** Optical and biochemical markers of the liver tissue energy “reserve” and oxidative state.

BDL Time	Optical Parameters			Biochemical Parameters	
	NAD(P)H_total_	NAD(P)H_free_/NAD(P)H_bound_^a^	Redox Ratio ^b^	ATP/ADP ^c^	TBARS (nmol/mg prot) ^c^
Sham-operated	56.23 ± 3.65	3.00 ± 0.18 ^a^	0.070 ± 0.003	3.85 ± 0.90	0.19 ± 0.02
*t* = 72 h	51.44 ± 2.62	4.01 ± 0.21	0.097 ± 0.005	1.24 ± 0.31	0.31 ± 0.01

Values reported as means ± S.E., *n* = 5 livers per group. Statistical analysis, 72 h BDL versus sham-operated rats: ^a^, *p* < 0.01; ^b^, *p* < 0.005; ^c^, *p* < 0.001 within data in the same column. ATP/ADP: adenosine triphosphate/adenosine diphosphate; TBARS: thiobarbituric acid reactive substances.

## Supplementary Materials

Supplementary materials can be found at http://www.mdpi.com/1422-0067/19/9/2634/s1.

## Abbreviations

ADP	Adenosine diphosphate
AF	Autofluorescence
ALT	Alanine aminotransferase
AP	Alkaline phosphatase
AST	Aspartate transaminase
ATP	Adenosine triphosphate
a.u.	Arbitrary units
BDL	Bile duct ligation
CTAB	Hexadecyltrimethylammonium-bromide
GMG	Half-Gaussian modified Gaussian spectral functions
Lpgs	Lipopigments (Lipofuscin-like lipopigments)
MCD	Methyl choline deficient diet
NAD(P)H	Nicotinamide adenine dinucleotide (phosphate)
OMA	Optical multichannel analyzer
PBS	Phosphate-buffered saline
S.E.	Standard error
TBARS	Thiobarbituric acid reactive substances
γGT	γ-glutamyltransferase
