# Supplementary material for: Serum and Hepatic Autofluorescence as a Real-Time Diagnostic Tool for Early Cholestasis Assessment
Source: Int J Mol Sci. 2018 Sep 5;19(9):2634. doi: 10.3390/ijms19092634 (PMC6165295; doi:10.3390/ijms19092634)
Supplement: Supplementary file 1 [file ijms-19-02634-s001.pdf]

Supplementary Materials

Serum and Hepatic Autofluorescence as a Real-Time Diagnostic Tool for Early Cholestasis Assessment

Anna C. Croce [1], Giovanni Bottiroli [1], Laura Giuseppina Di Pasqua [2], Clarissa Berardo [2], Veronica Siciliano [2], Vittoria Rizzo [3], Mariapia Vairetti [2], Andrea Ferrigno [2,*]

[1] Institute of Molecular Genetics, Italian National Research Council (CNR), Via Abbiategrasso 207, and Department of Biology & Biotechnology, University of Pavia, Via Ferrata 9, I-27100 Pavia, Italy, croce@igm.cnr.it (A.C.C.); bottiroli@igm.cnr.it (G.B.)

[2] Department of Internal Medicine and Therapeutics, University of Pavia, Via Ferrata 9, I-27100 Pavia, Italy; lauragiuseppin.dipasqua01@universitadipavia.it (L.G.D.P.); clarissa.berardo01@universitadipavia.it (C.B.); veronica.siciliano01@universitadipavia.it (V.S.); mariapia.vairetti@unipv.it (M.V.), andrea.ferrigno@unipv.it (A.F.)

[2,3] Department of Molecular Medicine, IRCCS San Matteo, University of Pavia, Viale Golgi 5, I-27100 Pavia, Italy, v.rizzo@smatteo.pv.it (V.R.)

* Correspondence: andrea.ferrigno@unipv.it; Tel.: +39-0382-986-451

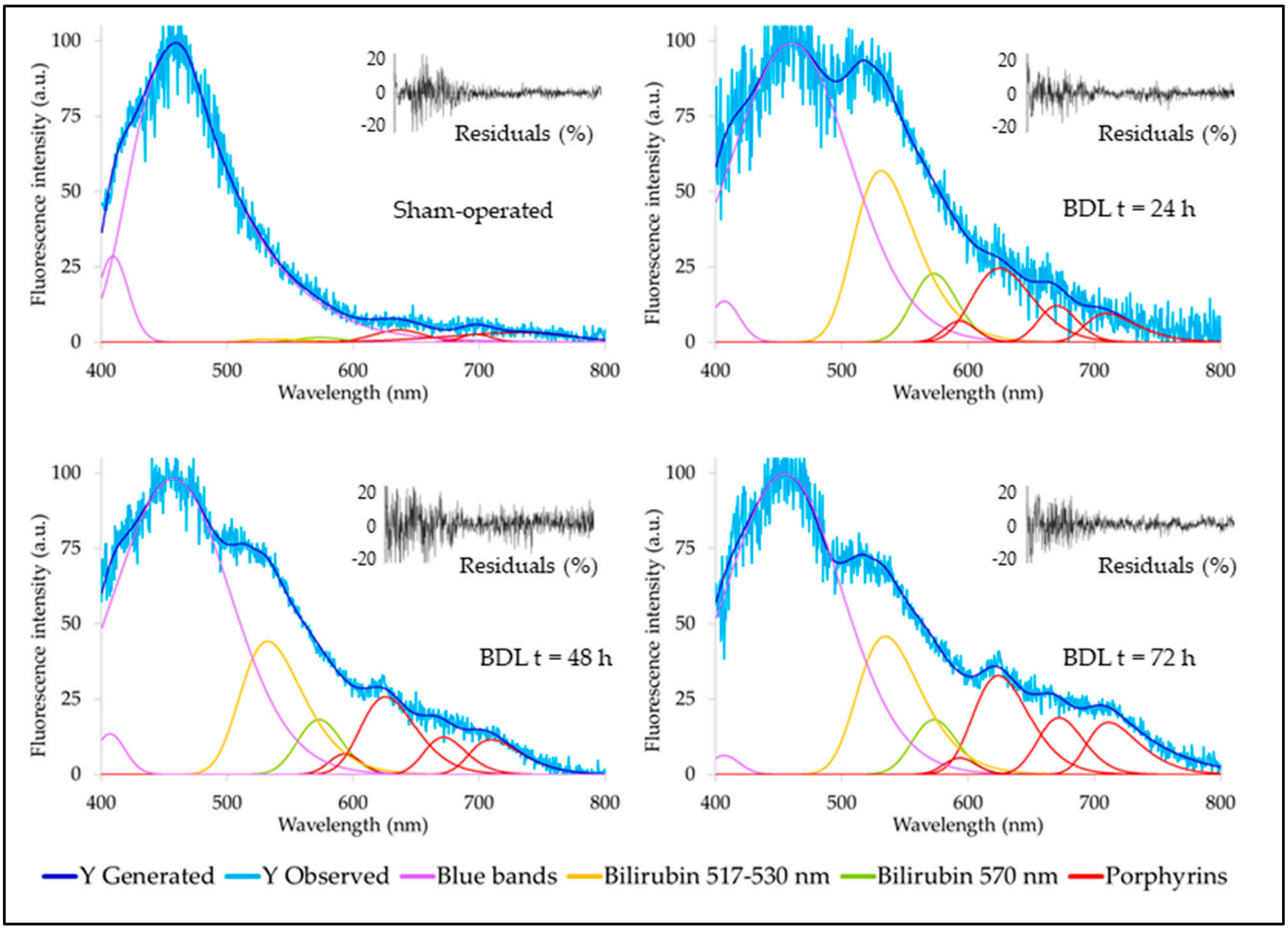

Figure S1. Examples of curve fitting analysis of the serum AF spectra from sham-operated or BDL rats. Fitting analysis is based on GMG functions, defined by the PeakFit program in terms of peak center wavelength (λ) and full width at half maximum (FWHM) parameters to match specifically with two bands ascribable to bilirubin (λ = 517–530 nm, FWHM 53 nm; λ = 570 nm, FWHM = 45 nm, [17]), three bands ascribable to porphyrins ($\lambda \approx$ 625 nm, FWHM ≈ 55 nm; $\lambda \approx$ 665 nm, FWHM ≈ 40 nm, $\lambda \approx$ 710 nm, FWHM ≈ 50 nm, [18]), to the main band in the blue region ($\lambda \approx$ 455–460 nm, FWHM ≈ 105 nm), or to additional minor bands ($\lambda \approx$ 405–410 nm; $\lambda \approx$ 590–600 nm), allowed to adapt freely so as to obtain a satisfying combination. The goodness of fitting was verified by residual analysis (insets) and coefficient of determination ($r^2 \geq 0.897$).

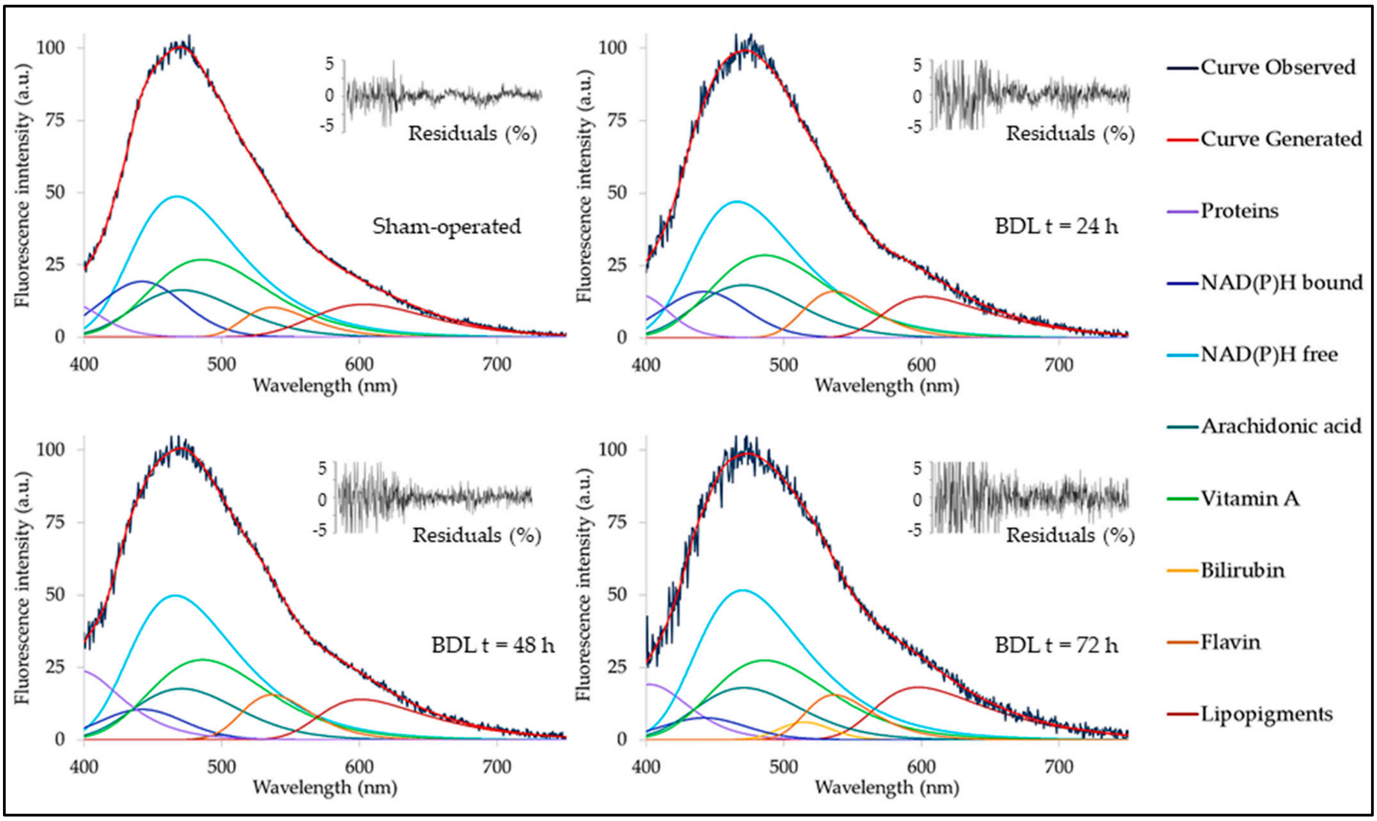

Figure S2. Examples of curve fitting analysis of the liver AF spectra from sham-operated or BDL rats. Fitting analysis is based on GMG functions, defined by the PeakFit program in terms of peak center wavelength (λ) and full width at half maximum (FWHM) parameters typical of each fluorophore in previous experiments [48]: NAD(P)H free λ = 463 nm; FWHM = 115 nm) and bound (λ = 444 nm; FWHM = 105 nm), flavins (λ = 526 nm; FWHM = 81 nm), vitamin A (λ = 488 nm; FWHM = 102 nm), arachidonic acid (fatty acids, λ = 470 nm; FWHM = 90 nm), proteins (emission tail, λ < 440 nm) and lipofuscin-like lipopigments λ 587 nm; FWHM = 80 nm). The GMG functions relatable to proteins and lipofuscin-like lipopigments were allowed to adapt freely so as to obtain a satisfying combination because of the variability of the emission of these components in accordance with their heterogeneous chemical composition, oxidation, and crosslink degrees.

The goodness of fitting was verified by residual analysis (insets) and coefficient of determination ($r^2 \geq 0.934$).
